# Molecular epidemiology of zoonotic streptococcosis/lactococcosis in rainbow trout (*Oncorhynchus mykiss*) aquaculture in Iran

**Published:** 2010-12

**Authors:** S Haghighi Karsidani, M Soltani, G Nikbakhat-Brojeni, M Ghasemi, HF Skall

**Affiliations:** 1 Department of Aquatic Animal Health, Faculty of Veterinary Medicine, University of Tehran.; 2 Department of Microbiology, Faculty of Veterinary Medicine, University of Tehran.; 3 Department of Aquatic Animal Health, Inland Water Aquaculture Institute, Bandar Anzali, Iran.; 4 Technical University of Denmark Hangøvej 28200, Århus N, Denmark.

**Keywords:** Streptococcosis, lactococcosis, rainbow trout, Iran

## Abstract

**Background and Objectives:**

Streptococcosis/lactococcosis is a hyperacute systemic disease that can occur in marine and fresh waters of many species of fish. The aim of this work was to study the disease outbreak in the major rainbow trout (*Oncorhynchus mykiss)* production of Iran.

**Materials and Methods:**

108 Gram positive cocci isolates were obtained from diseased trout in seven provinces with major trout production during 2008 till 2009. These bacterial isolates were characterized using phenotypic and molecular studies. The isolates were also analysed phylogeneticaly and compared with the available data.

**Results:**

49 samples (45.37%) were identified as *Streptococcus iniae*, 37 samples (35.2%) matched with *Lactococcus garvieae;* and 22 samples (19.43%) were identified as members of *Streptooccus* genus by culture-based and biochemical tests of API 50 CH, API 20 STREP and rapid 32 STREP systems. Using universal primers for differentiation of *Streptococcus sp*. and *Enterococcus sp*, all 108 samples were identified as *Streptococcus* sp. with a target region of 500 bp. Single specific PCR resulted in identification of 64 (59.2%) isolates as *S. iniae* and 44 (40.8%) isolates as *L. garvieae*. The phylogenetic analysis of the *S. iniae* isolates resulted in maximal similarity to some strains reported from Taiwan and to all Brazilian strains. Also, one strain showed less sequence similarity values with other tested strains although this strain has high similarity with ATCC 29178 strain, all reported Chinese, and some Taiwanian strains. Also, analysis of *S. iniae* LctO gene sequence showed that this isolate clustered within the *S. iniae* group. The sequence analysis of *L. garvieae* strains also showed that they have maximum similarity to all Japanese and Chinese strains, but one strain has lower sequence similarity values with all other recorded strains.

**Conculsion:**

The results of this study clearly show that trout farming in Iran is severely affected by both species of *S. Iniae* and *L. garvieae* and requires serious preventive criteria.

## INTRODUCTION

Streptococcosis/lactococcosis was described as a hyperacute systemic disease that can occur in marine and fresh waters of many species of fish including rainbow, tilapia, sea bass, eel and yellow tail (1–6). The disease also known as pop-eye disease, is now one of the most important bacterial diseases in farmed rainbow trout in almost all countries having trout aquaculture activity (1, 2, 7). Several species of *Streptococcus* and *Lactococcus* bacteria including *S. iniae, S. agalactiae, S. dysagalactiae, S. parauberis, S. feacalis, L. garvieae* and *L. lactis* have been so far discriminated as the cause of streptoococcosis/ lactococcosis (2, 4, 8–10).

Iran is now one of the leading countries in trout production in freshwater with a total production of about 60000 tons in 2008 (Iranian Fisheries Organization, 2008). Since the first reports of a presumptive streptococcosis (11), *S. iniae* and *L. garvieae* were identified as causative agents of the disease during 2005–2008 (12, 13). Despite significant losses due to this zoonotic bacterial disease in trout aquaculture in Iran, little information is available particularly on the epizootiology and the causative agents involved. In the present study, the disease epidemiology has been assessed in seven major trout- producing provinces. Conventional bacteriology and polymerase chain reaction (PCR) were used to compare the accuracy of disease detection. Also, isolated bacteria were phylogenetically characterized and compared with available data.

## MATERIALS AND METHODS

**Source of bacterial isolates**. Total of 108 isolates of Gram positive cocci bacteria were used. These bacterial isolates were obtained from farmed rainbow trout in seven provinces of Iran including Mazandran (36 isolates), Tehran (17 isolates.), Gilan (13 isolates), Kermanshah (2 isolates), Lorstan (14 isolates), Fars (18 isolates) and Charmahal- va-Bakhteyari (8 isolates). The bacterial isolates were recovered from kidney or spleen tissues of diseased fish on blood agar medium incubated at 25–30°C for 72 h. During the sampling time,clinical observations and water quality parameters were also recorded.

**Phenotypic characterization**. The pure colonies of fresh cultures were subjected to morphological and biochemical tests for phenotypic characterization (3, 14–16). Biochemical tests including acidification of carbohydrates and enzymatic tests were performed with API 50 CH, API 20 STREP and Rapid 32 STREP (Biomerieux, France). Manufacturers? instructions were followed except for the incubation temperature for API 50 CH and API 20 STREP, which was maintained at 24±1°C instead of the recommended 36 ± 1°C. Final results were read at 4 h for API Rapid 32 STREP and at 72 h for API 50 CH and API 20 STREP after incubation.

**Extraction of bacterial DNA**. DNA was extracted from pure colonies using the rapid genomic DNA isolation kit (MBST Company, Iran) based on extraction by proteinase K according to the manufacturer's instructions. Extracted DNA was dissolved in 100 µl of distilled water and stored at −20°C until used.

**PCR amplification of the** ***Streptococcus*** **sp. and** ***Enterococcus*** **sp. 16S rRNA gene**. Initially two pairs of universal primers of *Streptococcus* sp and *Entrococcus* sp were used to amplify the 16S rRNA gene for diagnosis of *Streptococcus* and *Entrococcus* genera (8, 17, 18) (Table 1). The PCR amplification were performed in 25 µl reaction mixture containing 1.5 µl of template DNA, 100 pmol concentration each primer (all primers were synthesized by DNA technology A/S, Arhus, Denmark), 2.5 mM MgCl, 10 mM concentration of each dNTP and 2U of Taq DNA polymerase (Promega, USA) in 5X reaction flexi buffer. After a denaturation step at 94°C for 5 min, 30 serial cycles consisting of a denaturation step at 92°C for 1 min, annealing at 55°C for 1 min, and extension at 72°C for 90 s were run followed with a final extension step at 72°C for 5 min. A negative control (no template DNA) and positive control consisting of *S. iniae* (ATCC29178) *L. garvieae* (TKS KG+), *S. parauberis* (NCDO2020), and *E. faecalis* strains (CCUG19916) were included in each run.

**Table 1:** Oligonucleotid primers used for single PCR assays.

Primer pairs	Sequence (5′-3′)	Target gene	PCR Amplicon (bp)	Pathogen	Reference
Strep.sp.	AGAGTTTGATCCTGGCTCAG	16S rRNA	500	*.Streptococcus sp*	Conrads et al., 1997
FW/BW	GTACCGTCACAGTATGAACTTTCC				
Entero.sp	TAC TGA CAA ACC ATT CAT GAT G	16S rRNA	112	*.Enterococcus sp*	Ke et al., 1999
FW/BW	AAC TTC GTC ACC AAC GCG AAC				
Spa2152	TTTCGTCTGAGGCAATGTTG	23S rRNA	718	*S. parauberis*	Riffon et al., 2001
Spa2870	GCTTCATATATCGCTATACT				
LOX-1	AAGGGGAAATCGCAAGTGCC	IctO	870	*S. iniae*	Mata et al., 2004
LOX-2	ATATCTGATTGGGCCGTCTAA				
PlG-1	CATAACAATGAGAATCGC	16S rRNA	1100	*L. garvieae*	Mata et al., 2004
PlG-2	GCACCCTCGCGGGTTG				
FW/BW (V1/V2)	V1:5′-TTTGGTGTTTACACTAGACTG-3′	16SrRNA	120	*S.agalactiae*	Meiri-Bendek et al., 2002
	V2: 5′-TGTGTTAATTACTCTTATGCG-3′				
FW/BW (Strd-dyl/Dys-16s-23s-2)	5′-TGGAACACGTTAGGGTCG-3′	16S-23S rDNA	300	*S.dysgalactiae*	Forsman et al.,1997; Hassan et al., 2003
	5′CTTAACTAGAAAAACTCTTGATTATTC-3′				

**PCR amplification of** ***S. iniae*** **lactate oxidase (lctO)**, ***S. parauberis*** **23 rRNA**, ***S. dysagalctiae*** **16S-23S rDNA and** ***S. agalactiae*** **16SrRNA and** ***L. garvieae*** **16S rRNA, genes**. The oligonucleotid primers used for PCR amplification of *S. iniae*, *L.garvieae*, *S. parauberis, S. agalactiae and S. dysagalactiae* genes are given in Table 1. At the first step all bacterial strains were subjected to PCR for identification of *S. iniae*. At the second step, those bacterial strains that were negative for *S. iniae* were subjected to PCR for identification of *L. garvieae*. Based on the results of phenotypic features, 22 bacterial strains showed variable reactions particularly to tested sugars. Therefore, these bacterial strains were also subjected to PCRs for *S. agalactiae* and *S. dysgalactiae*.

The PCR amplification for *S. iniae*, *L. garvieae* and *S. parauberis* were performed in 25 µl reaction mixture containing 1.5 µl of template DNA, 100 pmol concentration of each primer (all primers were synthesized by ISOGEN Bioscience BV, polymerase buffer (1.5 mM MgCl); 1.0 *µ*l of each forward and reverse primers (10 *µ*M each); 0.2 *µ*l of dNTP (25 mM), 0.1 *µ*l of Taq polymerase (0.25 u); 5 *µ*l of DNA (50 to 100 ng/*µ*l); add ddH2O (sterile) to total volume 25 *µ*l. The reaction was carried out in a PCR thermocycler as follows: 94°C for 4 min; five cycles of 94°C, Tm°C and 72°C for 45 s each step; 20 cycles of 94°C, 72°C for 45 s each step; and a step of 72°C for 5 min, at the end of the reaction. Also, to amplify part of the 16S–23S rDN intergenic spacer region that is specific to *S. dysgalactiae*, the oligonucleotide forward and reverse primers (dys-16S-23S-2) recommended by Forsman et al. (18) and Hassan et al. (19) were used (Table 1). The PCR assay was performed according to Hassan et al. (2003) using a thermal cycler (Biorad). PCR products were run on agarose gel (1.8 to 2.0%) and visualized by Etidium Bromide 0.005%. A negative control (no template DNA) and positive controls of these bacterial strains were also included in each PCR run.

**16S rRNA and lctO genes sequence analysis**. The PCR products of 16S rRNA of *L. garvieae* and lctO of *S. iniae* were sequenced. Sequencing of each PCR product was undertaken using DNA technology A/S analyzer. The forward and reverse nuclide acid sequence data were used to construct a continuous sequence of inserted DNA. Further comparison of the continuous sequences was then made with previously available sequences in the NCBI data base using BLAST (Basic Local Alignment Search Tool). Multiple sequence alignment analysis and construction of a phylogenetic tree were performed using MEGA 4 software via FASTA algorithms. The phylogenic trees were then constructed on the basis of the UPGMA method and the evolutionary distances were estimated using MEGA 4 (16). Maarssen, The Netherlands), 2.5 mM MgCl_2_,10 mM concentration of each dNTP and 5U of Taq DNA polymerase (Cinagene, Iran) in 10X reaction buffer. After a denaturation step at 94°C for 5 min,30 serial cycles consisting of a denaturation step at 92°C for 1 min, annealing at 58.6°C for *S. iniae*;52.7°C for *L. garvieae* and 52.5°C for *S. parauberis* for 1 min, and extension at 72°C for 90 s were used. The final extension step was performed at 72°C for 5 min. For *S. agalactiae* we used the PCR procedure recommended by Meiri-Bendek et al.(15). Brifely, the PCR reaction mixture contained 2.5 µl of 10 ×Taq

## RESULTS

**Clinical observations**. Sluggish movement, darkening of body, bilateral exophthalmia sometimes together with cataract and hemorrhage, abdominal distention and prolaps of anal area with hyperemia/ hemorrhage were observable in most affected fish. Also, accumulation of bloody fluids in abdominal cavity, hemorrhage in intestinal lumen, pale liver and precarditis (in brood fish) were seen in dissection examination. In most cases, the affected fish farms were using rivers as the main source of their water with water temperature in the range of 14-20°C****, dissolved oxygen of 6-8 mg/l, carbon dioxide of 4–15 mg/l, nitrite of 0.0–0.1 mg/l, and unionized ammonia of 0.06-0.1 mg/l.

**Biochemical features**. Biochemical analyses showed that 45.37% of bacterial isolates matched with *S. iniae* isolates i.e. positive reactions on the Esculin (ESC), pyrrolidonyl arylamidase (PYRA), β glucuronidase (ß GUR), L-leucine arylamidase (LAP), trehalose (TRE), starch (STA), sucrose (SUC), maltose (MAL), galactose (GAL), D-glucose (GLU), D-fructose (FRU), D-manose (MNE), arbutin (ARB), salicin (SAL), cellobiose (CEL) and N-acetyl glucosamine (NAG) tests and negative reactions on the Voges-Proskauer (VP), hippurate hydrolysis (HIP), α-galactosidase (α GAL), β-galactosidase (ß-GAL), N-acetyl-β-glucosaminidase (ß-NAG), glycyl- tryptophan-arylamidase (GTA), Lactose (LAC), L-arabinose (LARA), sorbitol (SOR), inulin (INU), cyclodextrin (CDEX), melibiose (MEL), tagatose (TAG), erythritol (ERY), D-arabinose (DARA), D-xylose (DXYL), L-xylose ( LXYL), adonitol (ADO), L-sorbose (SBE), rhamnose (RHA), dulcitol (DUL), inositol (INO), xylitol (XLT), D-turanose (TUR), D-lyxose (LYX), D-tagatose (D-TAG), D-fucose (D-FUC), L-fucose (L-FUC), L-arabitol (L-ARL), gluconate (GNT), 2-keto-gluconate (2KG), 5-keto- gluconate (5KG), α-methyl-D-glucoside (MDG) and glycerol (GLY) tests. In addition, variable reactions were observed on the arginin dihydrolase (ADH), β glucosidase (ß-GLU), alkaline phosphatase (PAL), β-manosidase (ß-MAN), ribose (RIB), mannitol (MAN), raffinose (RAF), glycogen (GLYG), D-arabitol (DARL), melezitose (MLZ), puliulane (PUL), amygdalin (AMY) and ß-gentiobiose (GEN) tests.

Also 35.2% of bacterial isolates matched with *L. garvieae* i.e****. positive reactions to VP, ESC, ADH, ß-GLU, ß-NAG, RIB, MAN, TRE, MAL, methyl-Bd glucopyranoside acidification (MßDG), TAG, GAL, GLU, FRU, MNE, AMY, ARB, SAL,CEL, GEN, GNT and NAG and negative reactions to HIP, PYRA, ß-GLU, α-GAL, PAL, ß-GAL, GTA, ß-MAN, LAP, RAF, LARA, SOR, GLYG, INU, DARL, CDEX, MLZ, PUL, MEL, ERY, DARA, DXYL, LXYL, ADO, SBE, RHA, DUL, INO, XLT, TUR, LYX, DFUC, LFUC, LARL, 2KG, 5KG, α-methyl-D- mannoside (MDM), MDG and GLY. However, some *L. garvieae* isolates displayed variable reactions to LAC, STA and SUC.

The remaining 22 isolates were phenotypically identified as the members of *Streptococcus* genus having variable reactions for some tested sugars (data not shown).

**Fig. 1:**
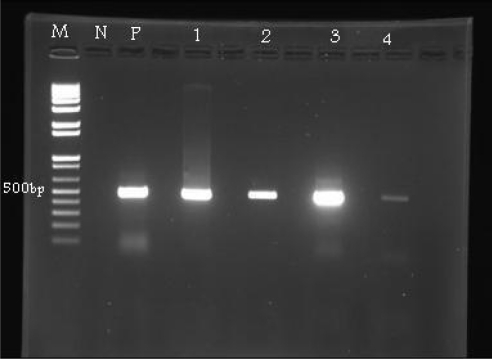
Representative amplification of PCR products using universal primers of *Streptococcus s*p. Lane M=Marker 100bp; Lane N=Negative control (*Entrococcus faecalis* CCUG19916); Lane P=positive control (*S. iniae*); Lanes 1, 2 and 3=test samples.

**PCR amplification of the** ***Streptococcus*** **sp. and** ***Enterococcus*** **sp. 16S Rrna**. A 500bp band was detected in all 108 bacterial isolates that confirms *Streptococcus sp*., while none of these samples revealed a 112bp band that is matched with *Enterococcus sp* (Fig. 1). Regional distribution of these isolates of *Streptococcus* sp. are given in Table 2. The highest and lowest infected trout farms were Mazandaran (33.3%) and Kermanshah (1.9%) regions, respectively.

**Table 2:** Regional distribution (%) of *L. garvieae, S. iniae* and Streptococcus sp based on traditional and molecular works in seven states with major trout production in Iran. Numbers in parentheses indicating the numbers of bacterial strains. LG=*L. garvieae, SI=S. iniae, SP=S. parauberis*, *SD=S. dysagalactiae, SA=S. agalactiae*.

State	Traditional bacteriology			PCR with universal primers		PCRwith specific primers				

	LG	SI	Strep. sp	Strept. sp	Ent. sp	LG	SI	SP	SA	SD
Gilan	0 (0)	8.2 (4)	40.9 (9)	12 (13)	0	0 (0)	20.3 (13)	0	0	0
Mazandran	29.7 (11)	38.8 (19)	27.3 (6)	33.3 (36)	0	39 (17)	29.7 (19)	0	0	0
Tehran	18.9 (7)	14.3 (7)	13.6 (3)	15.7 (17)	0	18.2 (8)	(9) 14	0	0	0
Kermansha	5.4 (2)	0 (0)	0 (0)	1.9 (2)	0	4.4 (2)	0 (0)	0	0	0
Charmahal va Bakhteyari	10.8 (4)	4.1 (2)	9.1 (2)	7.4 (8)	0	9 (4)	6.3 (4)	0	0	0
Lorestan	29.7 (11)	6.1 (3)	0 (0)	13 (14)	0	25 (11)	4.7 (3)	0	0	0
Fars	5.4 (2)	28.6 (14)	9.1 (2)	16.7 (18)	0	4.4 (2)	25 (16)	0	0	0
Total	100 (37)	100 (49)	100 (22)	100 (108)	0	100 (44)	100 (64)	0	0	0

**Fig. 2:**
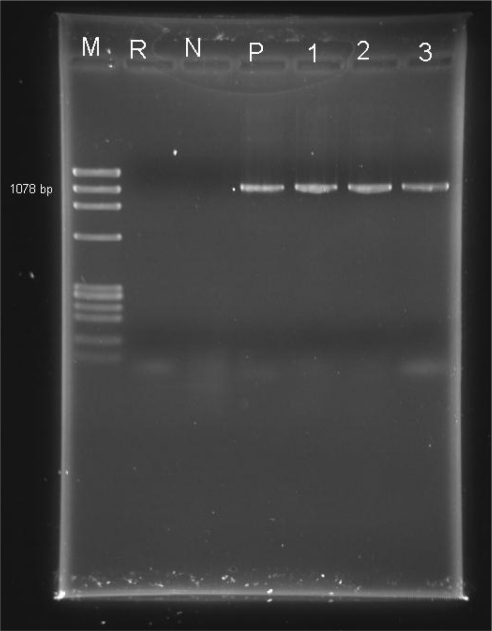
Amplification of the PCR products for detection of *L. garviae* (1100bp). Lane M=Marker; Lane B=distilled water; Lane N=Negative control (*S. iniae*); Lane P=Positive control (*L. garvieae*); Lanes 1, 2 and 3=test samples.

**Fig. 3:**
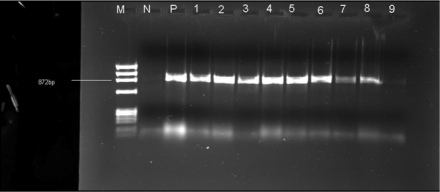
Amplification of the PCR products for detection of *S. iniae* (870bp). Lane M=Marker; Lane N=Negative control (*L. garvieae*); Lane P=Positive control (*S. iniae*); Lanes 1-9=test samples.

**Specific single pCRs amplification**. Each of the three pairs of primers exclusively amplified the targeted gene of the specific bacteria. From 108 bacterial isolates, 37 isolates showed 1100bp that is identical to *L. garviea* (Fig. 2) and 49 isolates revealed 870bp which is identical to *S. iniae* (Fig. 3). None of the samples produced a band of 718 bp which is identical to *S. parauberis*. The regional distribution of infection by *S. iniae* shows that trout farming in Mazandaran (29.7%) and Fars (25%) states were more affected than other examined states. Also, no infection by *S. iniae* was detected in Kermanshah region (Table 2). Furthermore, infection by *L. garvieae* was higher in Mazandran (39%) and Lorestan (25%) regions than other studied states, while no infection by *L. garvieae* was detected in Gilan region (Table 2).

**Sequence analysis**. The results of sequencing of the representative bacterial strains of *S. iniae* and *L.garvieae* showed 746bp and 856- 852bp, respectively****. These sequences were recorded in Gene bank under accession number ATCC numbers GQ850377, GQ850376, GQ850375, FJ870987 and HM055571-4 (Table 2).

**Fig. 4:**
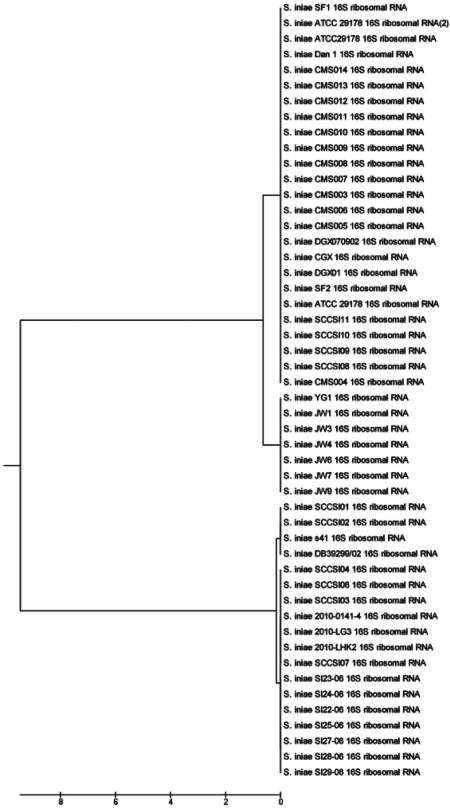
Phylogenetic tree based on 16S rRNA gene sequences constructed according to UPGMA method, showing the posi- tion of Iranian strains of S. iniae and the.isolates from other regions. (140×50 mm (400×400 DP).

**Fig. 5:**
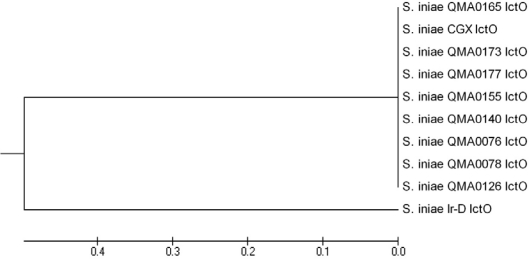
Phylogenetic tree based on lctO gene sequences, constructed according to UPGMA method, showing the position of Iranian strains of *S. iniae* and other isolates of S. iniae. 140×50 mm (400×400 DP).

**Phylogenetic analysis and genetic distance of S. iniae16s rRNA and lctO genes**. Partial 16S rRNA gene fragment of *S. iniae* strains LG3, LHK2, 0141 and SF2 were sequenced (Accession numbers HM055572, HM055573, HM055574 and FJ870987). Data for the phylogenetic analysis were obtained from sequences contained in the Gen Bank nucleotide sequences database. Strains LG3, LHK2 and 0141 from Iran have maximum similarity to strains SCC104, SCC106, SCC103 and SCC107 reported from Taiwan and all Brazilian strains. Lower sequence similarity values were found between strain SF2 and all other three Iranian strains. Strain SF2 has also high similarity with the ATCC 29178, all reported Chinese strains and some Taiwanian strains (Fig. 4). Also, LctO gene of the Ir-D strain was examined. Phylogenetic analyses inferred from LctO gene sequence comparisons using the neighbor-joining showed that Ir-D strain clustered within the *S. iniae* group (Fig. 5).

**Fig. 6:**
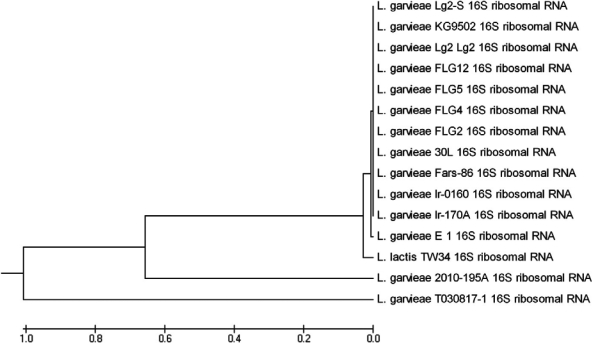
Phylogenetic tree based on 16S rRNA gene sequences constructed according to UPGMA method, showing the position of Iranian strains of *L. garvieae* and the isolates other regions. (140×50mm (400×400 DP).

**Phylogenetic analysis and genetic distance of** ***L. garvieae**.* A 856 bp 16S rRNA gene fragment of *L. garvieae* strains 195A, Ir-0160 and Ir-170A were sequenced (Accession numbers HM055571, GQ850376 and GQ850375). Phylogenetic tree for these strains along with the other reported sequences contained in the Gen Bank nucleotide sequences database is shown in Fig. 6. The Ir-0160 and Ir-170A strains have maximum similarity to all Japanese and Chinese strains, but strain 195A has lower sequence similarity values with all other recorded strains.

## DISCUSSION

Streptococcosis/lactococcosis have become one of the most serious bacterial pathogens causing significant losses in many farmed marine and freshwater fish species of both cold and warm water environments. Data obtained on the clinical observations as well as traditional and molecular bacteriology provides adequate information on the epizootiology of streptococcosis and lactococcosis outbreaks in trout aquaculture in Iran. Clinically, in most cases the affected farms showed a chronic to subacute disease and the most diseased fish showed bilateral exophthalmia together with cataract and in some cases complete loss of the eyes. Sluggish movement, darkening of body, mild abdominal distention, prolaps of anal area, hemorrhage in the intestine and accumulation of bloody fluid in the abdominal cavity were also clinically observable signs. Total mortality was varied from 5 to 50% during a period of approximately 3 month of fish farming. In most cases the affected fish were above 100 g. The water temperature of all affected farms were in the range of 13-19°C. The river water was the main source of water for those fish farms with more severe disease outbreaks.

Conventional bacteriology resulted in the isolation and characterization of 108 Gram positive cocci bacterial strains identical to *S. iniae* (45.35%) and *L. garvieae* (35.18%). The remaining isolates (19.43%) were identified as members of *Streptococcus* genus based on the phenotypic and PCR analysis using universal primers. When these bacterial isolates were subjected to the specific PCR analysis, about 60% of them were characterized as *S. iniae* and about 40% as *L. garvieae* ****. Therefore, these data show that *S. inae* and *L. garvieae* are the main causative agents of streptococcosis/lactocococcis outbreaks in the major trout production states of Iran.

For the phylogenic analysis and investigation of relationship between Iranian isolates and the other isolates in the world, we initially searched in NCBI and found several isolates of *S. iniae* in different regions such as Australia (8 strains), Brazil (7 strains), China (18 strains), Taiwan (10 strains) Singapore (1 strain), Thailand (6 strains), USA (1 strain) and Middle East (4 strains) (Table 3). Also, 10 isolates of *L. garvieae* were found in different countries including Japan (4 strains), China (5 strains) and Iran (1 strain). Result of sequencing of the representative strains of *S. Iniae* 16S rRNA gene shows that Iranian strains are closer to Taiwan and Brazilian strains than other reported strains (Fig. 3). Also, phylogenetic analysis of *S. iniae* LctO gene shows that the Iranian strains are clustered within *S. iniae* group having more genetic distance with other recorded strains reported from other regions (Fig. 4). In addition, the sequencing of the representative strains of *L. garvieae* shows that Iranian strains are closer to isolates reported from both China and Japan than other strains (Fig. 5). However, strain 195A showed lower similarity values to other reported strains.

**Table 3:** Data on *S. iniae* and *L. garvieae* strains analyzed in phylogenic analysis.

**Country**	**Bacterial species**	**Accession number**	**Source (year)**	**Target gene**
Argentina	*L. garvieae* TW34 (1398bp)	GQ845022	*Odontesthes platensis* (2010)	16S rRNA
Australia	*S. iniae* QMA0078 (1180 bp)	EU086698	*Lates calcarifer* (2007)	LctO
	*S. iniae* QMA0076 (1228bp)	EU086697	*L. calcarifer* (2007)	LctO
	*S. iniae* QMA00126 (1279bp)	EU086699	*L. calcarifer* (2007	LctO
	*S. iniae* QMA00140 (1228bp)	EUO86700	*L. calcarifer* (2007)	LctO
	*S. iniae* A00155 (1228bp)	EUO86701	*L. calcarifer* (2007)	LctO
	*S. iniae* QMA00177 (1248bp)	EU086704	*L. calcarifer* (2007)	LctO
	*S. iniae* QMA00173 (1185bp)	EU086703	*L. calcarifer* (2007)	LctO
	*S. iniae* QMA00165 (1176bp)	EU086702	*L. calcarifer* (2007)	LctO
Brazil	*S. iniae* S122-06 (398bp)	FJ803994	*Oreochromis niloticus* (2010)	16S rRNA
	*S. iniae* S123-06 (522bp)	FJ803995	*O. niloticus* (2010)	16S rRNA
	*S. iniae* S124-06 (522bp)	FJ803996	*O. niloticus* (2010)	16S rRNA
	*S. iniae* S125-06 (702bp)	FJ803997	*O. niloticus* (2010)	16S rRNA
	*S. iniae* S127-06(696bp)	FJ803998	*O. niloticus* (2010)	16S rRNA
	*S. iniae* S128-06 (500bp)	FJ803999	*O. niloticus* (2010)	16S rRNA
	*S*. iniae S129-06 (688bp)	FJ804000	*O. niloticus* (2010)	16S rRNA
Chile	*L. garvieae* 30L (817bp)	FJ151399	Aplodactylus punctatus (2008)	16S rRNA
China	*S. iniae* CGX (870bp)	EF126045	Tilapia (2006)	
	*S. iniae* CGX (1447bp)	DQ985468	Tilapia (2006)	LctO
	*S. iniae* SF1 (501bp)	GQ891547	*Japanese flunder* (2010)	16S rRNA
	*S. iniae* DGX01 (1500bp)	HM053435	Channel cat fish (2010)	16S rRNA
	*S. iniae* YG1 (1168bp)	GQ169798	*Selenotoca multifasciata* (2009)	16S rRNA
	*S. iniae* DGX070902 (1497bp)	FJ951434	Channel cat fish (2009)	16S rRNA
	*S. iniae* CMS004 (1465bp)	EU620577	Unknown fish (2008)	16S rRNA
	*S. iniae* CMS005 (1463bp)	EU620578	Unknown fish (2008)	16S rRNA
	*S. iniae* CMS006 (1464bp)	EU620579	Unknown fish (2008)	16S rRNA
	*S. iniae* CMS003 (1464bp)	EU620580	Unknown fish (2008)	16S rRNA
	*S. iniae* CMS007 (1464bp)	EU622508	Unknown fish (2008)	16S rRNA
	*S. iniae* CMS008 (1464bp)	EU622509	Unknown fish (2008)	16S rRNA
	*S. iniae* CMS009 (1463bp)	EU622510	Unknown fish (2008)	16S rRNA
	*S. iniae* CMS0010 (1463bp)	EU622511	Unknown fish (2008)	16S rRNA
	*S. iniae* CMS0011 (1463bp)	EU622512	Unknown fish (2008)	16S rRNA
	*S. iniae* CMS0012 (1463bp)	EU622513	Unknown fish (2008)	16S rRNA
	*S. iniae* CMS0013 (1463bp)	EU622514	Unknown fish (2008)	16S rRNA
	*S. iniae* CMS0014 (1463bp)	EU622515	Unknown fish (2008)	16S rRNA
	*L. garvieae* T030817-1 (1427bp)	DQ010113	*Paralichthys olivaceus* (2005)	16S rRNA
	*L. garvieae* FLG2 (1544bp)	AF352163	*Mugil cephalus* (2002)	16SrRNA
	*L. garvieae* FLG4 (1544bp)	AF352164	*M. cephalus* (2002)	16SrRNA
	*L. garvieae* FLG5 (1544bp)	AF352165	*M. cephalus* (2002)	16SrRNA
	*L. garvieae* FLG12 (1544bp)	AF352166	*M. cephalus* (2002)	16SrRNA
Iran	*S. iniae* Ir-D (746bp)	GQ850377	*Oncorhynchus mykiss* (2009)	LctO
	*S. iniae* SF2 (1384bp)	FJ870987	*O. mykiss* (2009)	16SrRNA
	*S. iniae* 0141-4 (344bp)	HM055574	*O. mykiss* (2010)	16SrRNA
	*S. iniae* LHK2 (369bp)	HM055573	*O. mykiss* (2010)	16SrRNA
	*S. iniae* LG3 (407bp)	HM055572	*O. mykiss* (2010)	16SrRNA
	*L. garvieae* 195A (409bp)	HM055571	*O. mykiss* (2010)	16SrRNA
	*L. garvieae* Ir-170A (856bp)	GQ850376	*O. mykiss* (2009)	16SrRNA
	*L. garvieae* Ir-0160 (852bp)	GQ850375	*O. mykiss* (2010)	16SrRNA
	*L. garvieae* Fars (1007bp)	EU727199	*O. mykiss* (2008)	16SrRNA
Israel	*S. iniae* Dan1 (1490bp)	AF335573	*O. mykiss* (2001)	16SrRNA
	*S. iniae* S41 (654bp)	AY260834	*O. mykiss* (2003)	16S rRNA
	*S. iniae* ATCC29178 (1536bp)	AF335572	*O. mykiss* (2001)	16S rRNA
	*S. iniae* ATCC29178 (1536bp)	NR-025148	*O. mykiss* (2009)	16S rRNA
Japan	*L. garvieae* E1 (1506bp)	AB018211	*Cyprinus carpio* (2000)	16S rRNA
	*L. garvieae* Lg2 (1471bp)	AB267897	*Seriola quinqueradiata* (2006)	16S rRNA
	*L. garvieae* Lg2-S(1471bp)	AB267898	*S. quinqueradiata* (2006)	16S rRNA
	*L. garvieae* KG9502 (1471bp)	AB267899	*S. quinqueradiata* (2006)	16S rRNA
Singapore	*S. iniae* DB39299/02 (533bp)	DQ193527	Red tilapia (2005)	16S rRNA
Taiwan	*S. iniae* SCCS101 (510bp)	AY465111	*Rachycentron canadum* (2004)	16S rRNA
	*S. iniae* SCCS102 (517bp)	AY480053	*R. canadum* (2004)	16S rRNA
	*S. iniae* SCCS103 (513bp)	AY480054	*R. canadum* (2004)	16S rRNA
	*S. iniae* SCCS104 (506bp)	AY737430	R*. canadum* (2005)	16S rRNA
	*S. iniae* SCCS106 (497bp)	AY737432	*R. canadum* (2005)	16S rRNA
	*S. iniae* SCCS107 (497bp)	AY737433	*R. canadum* (2005)	16S rRNA
	*S. iniae* SCCS108 (497bp)	AY737434	*R. canadum* (2005)	16S rRNA
	*S. iniae* SCCS109 (497bp)	AY737435	*R. canadum* (2005)	16S rRNA
	*S. iniae* SCCS110 (522bp)	AY489403	*R. canadum* (2004)	16S rRNA
	*S. iniae* SCCS111 (520bp)	AY489404	*R. canadum* (2004)	16S rRNA
Thailand	*S. iniae* JW1 (1120bp)	GQ169769	*Oreochromis niloticus* (2009)	16S rRNA
	*S. iniae* JW3 (1130bp)	GQ338313	*O. niloticus* (2009)	16S rRNA
	*S. iniae* JW4 (1118bp)	GQ169770	*O. niloticus* (2009)	16S rRNA
	*S. iniae* JW6 (1120bp)	GQ338314	*O. niloticus* (2009)	16S rRNA
	*S. iniae* JW7 (1114bp)	GQ169771	*O. niloticus* (2009)	16S rRNA
	*S. iniae* JW9 (1141bp)	GQ338315	*O. niloticus* (2009)	16S rRNA
USA	*S. iniae* ATCC29178 (534bp)	AY577823	Unknown fish (2004)	16S rRNA

According to the geographical distribution of the identified bacterial strains shown in Table 2, it is clear that the trout farming in the states of Mazandran, Tehran, Charmahal-va-Bakhteyri, Lorstan and Fars are affected with both species of *S. iniae* and *L. garvieae*, while fish farms of Gilan and Kermanshah regions are infected with either *S. iniae* or *L. garvieae*. Also, it seems that infection by *S. iniae* is more dominant in Fars region than other investigated areas, while the outbreaks by *L. garvieae* was more in Lorestan state. In previous studies by Soltani et al. (13, 19), infections by either *S. inaie* or *L. garvieae* was detected as the cause of the disease outbreak in some trout farming in Iran. However, it is possible that infection by other members of *Streptococcus* genus may be involved in some farmed trout located in other regions of the country and therefore, warranted further investigations.

Most of disease outbreaks were detected during the warm seasons, late spring till mid autumn, and the time that water temperature of trout farming increases up to 20C particularly in those fish farms that use rivers as the source of water. Increase in water temperature together with impact of polluted water sources will cause a significant decline in water quality parameters resulting in outbreaks by infectious diseases including streptococcosis/lactococcosis.(1, 2, 10).

It is notable that the owners of most affected fish farms have no adequate training related to health management criteria. Such training is nowadays; very important particularly in the case of streptococcosis/ lactococcosis that is a human and terrestrial animal zoonotic disease (1, 2, 10, 20), providing it easy transportation to the fish farms through sewage of terrestrial animals. In Iran, transportation of eyed- eggs, larvae and broodstock between the fish farms is currently undertaken by many trout hatcheries providing an easy way for disease spreading inside the country.

Annual losses by streptococcosis/lactococcosis has been estimated at 100 million USD. In fact, the economic impact due to this bacterial disease is quite higher than 100 million USD. For instance, Iran is one of the leading countries in production of trout in freshwater having above 60000 ton per year and our annual estimated losses due to this zoonotic disease is about 15 million USD (26). This is a reason why the Iran veterinary organization has recently established the national committee of trout streptococcosis to reduce losses due to this highly devastating zoonotic contagious disease.

In conclusion, clinical observations plus molecular studies show that both S*. iniae* and *L. garvieae* are the causative agents involved in disease outbreaks in major trout production regions of Iran. Also, some other members of *Streptococcus* sp may be involved in disease production in Iranian trout aquaculture. Therefore, further investigations are warranted. Also, poor water quality, high water temperature and poor health management criteria, e.g. quarantine and other protective measures such as lack of vaccination, are the main reasons for disease spread inside the country.
